# Differences in Transdiaphragmatic Pressure of Dogs Suffering from Cervical or Thoracolumbar Myelopathy Anaesthetised with Isoflurane †

**DOI:** 10.3390/ani15020147

**Published:** 2025-01-09

**Authors:** Eirini Sarpekidou, Kyriaki Pavlidou, Ioannis Savvas, Zoe Polizopoulou, George Kazakos

**Affiliations:** 1 Surgery & Obstetrics Unit, Companion Animal Clinic, School of Veterinary Medicine, Faculty of Health Sciences, Aristotle University of Thessaloniki, 54627 Thessaloniki, Greece; 2 Anaesthesia and Intensive Care Unit, Companion Animal Clinic, School of Veterinary Medicine, Faculty of Health Sciences, Aristotle University of Thessaloniki, 54627 Thessaloniki, Greece; kpavlido@vet.auth.gr (K.P.); isavas@vet.auth.gr (I.S.); gkdvm@vet.auth.gr (G.K.); 3 Diagnostic Laboratory, Companion Animal Clinic, School of Veterinary Medicine, Faculty of Health Sciences, Aristotle University of Thessaloniki, 54627 Thessaloniki, Greece; poliz@vet.auth.gr

**Keywords:** trans-diaphragmatic pressure (Pdi), cervical myelopathy, thoracolumbar myelopathy, focal myelopathy, dogs, isoflurane

## Abstract

Background: The purpose of this study is to evaluate differences in transdiaphragmatic pressure (Pdi) in dogs suffering from cervical myelopathy (CM) and thoracolumbar myelopathy (TLM) anaesthetised with isoflurane (ISO). Methods: This prospective cohort study included ASA II-status dogs diagnosed with either CM or TLM, anaesthetised for diagnostic or surgical purposes between September 2021 and July 2023. Patients followed the same anaesthetic protocol, and maximum Pdi was recorded at 10 (Pdimax10), 20 (Pdimax20), and 30 (Pdimax30) minutes using balloon catheters placed in the stomach (Pgast) and in the mid-third of the oesophagus (Poes). Results: A total of 50 dogs were included: 25 in the CM group and 25 in the TLM group. In the TLM group, Pdimax10 was 9.4 mmHg ± 6.1, and in the CM group, it was 7.2 mmHg ± 4.6 (*p* = 0.167). Pdimax20 was 10.2 mmHg ± 5.8 in the TLM group and 8 mmHg ± 5.1 in the CM group (*p* = 0.155). Pdimax30 was 9.2 mmHg ± 5.5 in the TLM group and 8.1 mmHg ± 5.5 in the CM group (*p* = 0.479). Conclusions: The findings suggest that diaphragmatic contractility is similar in dogs with CM and TLM under ISO anaesthesia.

## Simple Summary

Respiratory integrity of dogs under general anaesthesia results from the seamless contraction of the main respiratory muscle, the diaphragm, which is innervated by the phrenic nerve, which originates from the fifth, sixth, and seventh cervical segments. Diaphragmatic contractility can be assessed by transdiaphragmatic pressure measurement, a reliable method in dogs. Respiratory distress in dogs suffering from cervical focal myelopathy is reported in the veterinary literature, but it is unknown whether cervical focal myelopathy results in diaphragmatic contractility impairment. The aim of this study is to quantify transdiaphragmatic pressure in dogs under isoflurane anaesthesia affected by cervical myelopathy, compare the results with dogs affected by thoracolumbar myelopathy, and determine whether focal spinal disorders affect respiratory function. There appears to be a diaphragmatic contractility impairment in both cervical and thoracolumbar myelopathy, since there is a similar reduction of the transdiaphragmatic pressure in both conditions. These results could improve the quality, treatment strategies, and monitoring offered by anaesthesiologists and clinicians treating dogs suffering from focal myelopathies in the cervical and thoracolumbar spinal cord.

## 1. Introduction

The diaphragm, the primary respiratory muscle, separates the thoracic and abdominal cavities and contracts rhythmically to draw air into the lungs [1]. This contraction, assisted by the intercostal muscles, increases the volume of the thoracic cavity. The thoracic cavity increases in volume during inspiration and decreases during expiration. The intra-thoracic pressure during inspiration is lower than the atmospheric pressure. At the time of the glottis opening, the intrathoracic and atmospheric pressures equalise. During inspiration, reduced intra-thoracic pressure causes the small airways and pulmonary alveoli to expand, allowing oxygenation by the incoming air [2].

The contraction of the diaphragm expands the thoracic cavity and lungs. Diaphragmatic contractility can be measured through trans-diaphragmatic pressure (Pdi) measurements in human and dogs [3,4]. Additionally, the intercostal muscles, located between the ribs, play a vital role in the seamless functioning of the respiratory system in dogs. The anatomical and functional integrity of these muscles is crucial for normal respiration [1].

The respiratory centres located in the pons and medulla oblongata transmit neural impulses caudally through the cervical and thoracic spinal cord to motor neurons. These motor neurons innervate the diaphragm and intercostal muscles, which serve as essential respiratory muscles. The phrenic nerves, which are the sole motor nerves of the diaphragm, arise from the cervical segments C5, C6, and C7 (occasionally C4) and are divided into left and right branches innervating each side of the diaphragm [1,5]. Neural impulses are also transmitted from the respiratory centres to all thoracic nerves. The ventral branches (rami ventrales) of the thoracic nerves (except the thoracic nerves of T1 and T13), emerging from each thoracic spinal foramen, are the intercostal nerves that innervate the intercostal muscles, which facilitate normal respiration [1,5,6,7].

In dogs with CM caused by compressive or non-compressive underlying conditions such as IVDD, fibrocartilaginous embolism, vertebral fractures, and similar issues, respiratory distress may arise due to suspected paresis or paralysis of the diaphragmatic or intercostal muscles [1,6]. Such distress may arise from central or peripheral nervous system lesions leading to diaphragmatic contractility impairments and reducing thoracic wall expansion. Impaired chest cavity expansion leads to hypoventilation as tidal volume decreases, causing hypercapnia [2,8].

An indicator of the severity of hypoventilation in dogs is the values of PaCO2 in arterial blood samples. Elevated PaCO2 levels are associated with the need for mechanical ventilation [2,9]. Lesions located anterior to the C3 segment may cause acute respiratory distress or death by affecting the respiratory centres in the pons and medulla oblongata [6,9,10,11]. Damage to C5, C6, and C7 segments can result in severe respiratory distress, often necessitating mechanical ventilation. In human medicine, respiratory distress is a primary concern for patients with lesions located in the cervical spinal segments [12,13].

Clinical manifestations of respiratory distress may be impacted by the location of a spinal cord lesion in relation to the diaphragmatic innervation. Experimental studies in mice indicate that contralateral diaphragm paresis/paralysis can result in mild respiratory distress [13,14,15]. Acute lesions leading to spinal cord compression or injury can cause bilateral diaphragmatic paralysis, while peripheral nerve damage, invasive masses, or surgical injuries may cause contralateral paralysis [9,16,17].

Disturbed diaphragm innervation may worsen respiratory depression during anaesthesia. Anaesthesia is essential for the management and diagnostic procedures of dogs suffering from focal neurological conditions [2]. The intercostal muscles’ contraction is insufficient in dogs under general anaesthesia; thus, ventilation is primarily dependent on diaphragmatic contraction. Respiratory function can be assessed by measuring PaCO2 and PaO2 in arterial blood samples collected from awake and anaesthetised animals. In anaesthetised animals, Pdi measurements provide insight into respiratory function [2,4,9]. Pdi measurement is a non-invasive and reliable method for assessing diaphragmatic function [4,18,19].

In terms of human medicine, one can access the extensive literature regarding the relationship between CM and respiratory function [12,20,21,22]. However, the veterinary literature on this topic is moderate [9,16,23,24]. There is scarce information about the role of the diaphragm in respiratory distress in dogs with CM [25]. It is reported that mechanical ventilation may be required in dogs with CM [9,24,26]. Beyond the underlying pathology causing CM, neurosurgical procedures can also increase the risk of hypoxia and hypoventilation during anaesthesia. A dorsal surgical approach aiming spinal decompression or spinal stabilisation is more likely to require mechanical ventilation compared to a ventral approach a finding probably attributed to the fact that this surgical procedure is elected in more severe and complicated cases [9].

The purpose of the present study was to compare the frequency and severity of diaphragmatic contractility impairment between dogs with focal CM and dogs with focal TLM by assessing Pdi. Additionally, the association of neurological grading (MFS) with Pdi and respiratory function in dogs with CM and TLM were evaluated [27].

Respiratory function, as assessed by Pdi, in dogs with focal CM (C1–T2) was expected to differ from that of dogs with focal TLM (T3–L3). Pdi values were anticipated to be lower in CM dogs compared to TLM dogs due to diaphragmatic contractility impairment caused by direct phrenic nerve involvement. Higher neurological grading (MFS) in both CM and TLM dogs was expected to correlate with lower Pdi values, reflecting more severe respiratory impairment.

To our knowledge, there are no published prospective clinical studies regarding differences between the Pdi of dogs suffering from CM and TLM and the association between neurological grading (MFS) and Pdi values.

## 2. Materials and Methods

For the present prospective cohort study, approval was obtained from the Ethics Committee of the School of Veterinary Medicine, Aristotle University of Thessaloniki, Greece (Research Protocol No. 770, 2 September 2021). All owners were informed about the study and provided written consent.

### 2.1. Study Population

Dogs presented in the Surgery and Obstetrics Unit of Companion Animal Clinic of Aristotle University of Thessaloniki with neurological deficits associated with focal CM or TLM were included in the present study. Inclusion criteria were between 1 and 11 years of age and body weight within the normal range for their breed and age. After clinical examination and laboratory and imaging evaluation (plain radiographs of the thoracic and abdominal cavity and abdominal ultrasonography), all dogs were assigned an American Society of Anesthesiologists (ASA) physical status grading. Any dog that had an ASA status higher than ASA II was excluded from the study. According to the neurological examination and radiological findings of the spine, the animals were allocated into two groups (group CM and group TLM) depending on whether a C1–T2 or T3–L3 spinal cord defect was present, respectively. Additionally, dogs that were overweight or exhibited thoracic or abdominal pathology were excluded from the study.

### 2.2. MFS Grading

Every dog was assigned a grading according to Modified Franken Scoring by the clinician (ES) before anaesthesia [27]. In the absence of neurological deficits (grade 0/5) the dogs were excluded from the study. When pain was the only symptom on presentation, the dogs were graded as 1/5; when ataxia was present, as 2/5; in cases with severe ataxia and paresis, as 3/5; non-ambulatory dogs with intact deep pain perception were graded as 4/5; and in cases without deep pain perception, dogs were graded as 5/5.

### 2.3. Anaesthetic Protocol

Premedication consisted of intramuscular (IM) administration of dexmedetomidine (Dextomidor 0.5 mg/mL, Orion Pharma, Espoo, Finland) (DEX) at a dose of 180 μg m^−2^. Twenty minutes later, an intravenous (IV) catheter suitable for the size of the dog was placed in the cephalic vein.

Anaesthesia was induced with IV administration of Propofol (PPF) (Propofol MCT/LCT/Fresenius 1% 10 mg/mL, Fresenius Kabi, Graz, Austria) at an initial dose of 1 mg/kg, followed by additional doses of 0.5 mg kg^−1^ (to effect) until successful intubation was achieved. Maintenance of anaesthesia was accomplished using isoflurane (Iso-Vet 1000 mg/g Piramal Critical Care B.V., Voorschoten, The Netherlands) (ISO) in 100% oxygen. Anaesthesia was maintained with an end-tidal isoflurane (ISO) concentration of 1.5%, which was deemed appropriate based on assessment of reflexes and responses to noxious stimulation. Patients were allowed to breathe spontaneously throughout the procedure. However, if the end-tidal CO2 (ETCO2) exceeded 55 mmHg, mechanical ventilation would be initiated, and volume control ventilation with a tidal volume of 10 mL/kg and a respiratory rate adjusted to maintain an ETCO2 within the range of 35–45 mmHg would be used. The entire anaesthetic process was conducted by the same anaesthetist (KP).

Dogs received Lactated Ringer’s solution, and during all the diagnostic and surgical procedures, they were connected to a multiparameter monitor (Mindray uMEC12 Vet) to continuously monitor and record electrocardiography, respiratory rate, SpO2, ETCO2, rectal temperature, and oscillometric blood pressure (NIBP) measurements.

### 2.4. Procedure

**Figure 1:**
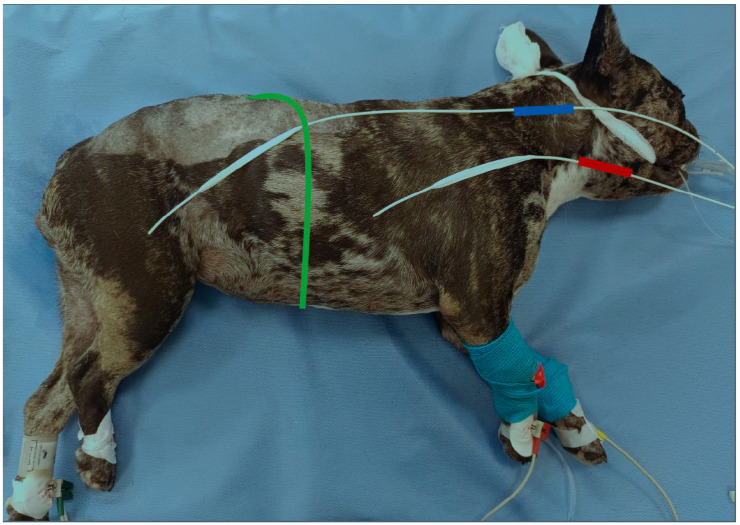
Indicative depiction of the catheter positions in the mid-third of the oesophagus (catheter marked in red) and the stomach (catheter marked in blue) in a dog. The green line represents the diaphragm.

All dogs in CM group and TLM group were positioned in the left lateral recumbency and remained in this position throughout the Pdimax measurements. The procedure began once the anaesthetist (KP) determined that the anaesthetic plane was adequate. Two ballon catheters (Adult esophageal ballon catheter, Cooper Surgical, Trumbull, CT, USA) were introduced orally for pressure measurements by the same clinician (ES). One catheter was placed into the stomach in order to measure the gastric pressure (Pgast), and the second catheter was placed in the mid-third of the oesophagus for measuring the oesophageal pressure (Poes) (Figure 1). The external end of each catheter was connected to a pressure transducer (Pressure Monitoring System Buzzer-II Michael Roehrich, Vienna, Austria), converting the pressure into digital signals, and the pressure measurements were displayed on a computer. Proper catheter placement was confirmed by observing the pressure waveforms; during inspiration, Pgast was positive, and Poes was negative. Once the correct placement was confirmed, the catheters were secured to the endotracheal tube, and 0.5–1 mL of air was injected into the balloon of each catheter.

Pdimax was evaluated and recorded at three different time points with an interval of 10 min. The catheters remained there for 30 min, and the maximum values of Pdimax (PGast − Poes) were recorded at 10 min (Pdimax10), 20 min (Pdimax20), and 30 min (Pdimax30) after placement. To measure the Pdimax, Mueller’s manoeuvre was applied by disconnecting the dog from the breathing circuit and blocking the endotracheal tube with the thumb, thus forcing the dog to breathe against a closed airway for three respiratory cycles.

The anaesthesia time did not exceed 60 min to allow for the completion of the anaesthetic protocol and the Pdi measurements.

**Figure 2:**
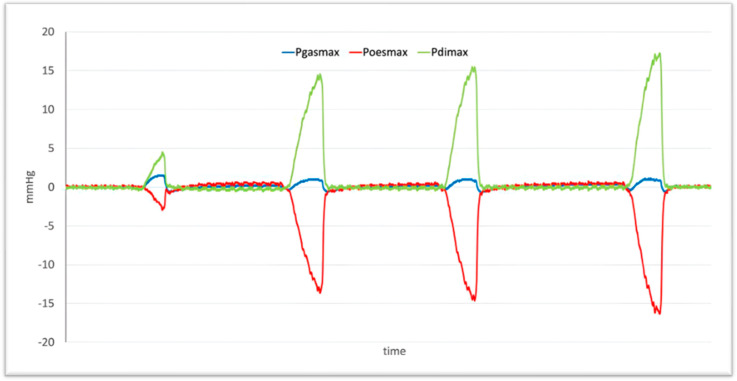
Line graph of four consecutive breathing cycles of a dog. The first cycle represents the pressures of a normal respiratory cycle under general anaesthesia. The following three cycles represent the pressures after the application of Mueller’s manoeuvre. The green line represents Pdimax (Transdiaphragmatic Pressure), the blue line indicates Pgasmax (Gastric Pressure), and the red line indicates Poesmax (Oesophageal Pressure).

Additionally, an arterial sample was collected for PaO2 and PaCO2 evaluation before starting (PaO2_0_ and PaCO2_0_) and after finishing (PaO2_30_ and PaCO2_30_) Pdimax measurements. The recorded data were analysed with a graphics analysis software (Origin Pro 7.5). The data were plotted on x-y graphs, and the Pdimax values for each respiratory cycle were identified (Figure 2). Then, the average of the three values at each time point was calculated.

### 2.5. Statistical Analysis

A power analysis was conducted, establishing a minimum of 20 dogs per group to achieve statistically significant results. All data were evaluated for normality with the Shapiro–Wilk normality test. A general linear model for repeated measures with one between-subject factor (group) and one within-subject factor (time) was used to evaluate any significant differences in the Pdimax. The measurements of the Pdimax at the three time points were averaged to produce one Pdimax value, which then was used in a regression analysis to evaluate any correlation between the Pdimax and the MFS. Statistical significance level was set to α < 0.05. For all statistical calculation, a dedicated software was used (IBM SPSS Statistics 29).

## 3. Results

### 3.1. Demographics

This prospective cohort study included a total of 50 client-owned dogs (n = 50) anaesthetised using a standardized anaesthetic protocol for radiological examinations or surgical interventions related to spinal cord disease. The study was conducted between September 2021 and July 2023.

The breeds were French bulldog, 15/50 (30%); mongrel, 14/50 (28%); cocker spaniel, 4/50 (8%); Maltese, 4/50 (8%); Pekingese, 3/50 (6%); Chihuahua, 2/50 (4%); pinscher, 2/50 (4%); Jack Russell, 2/50 (4%); beagle, 1/50 (2%); West Highland terrier, 1/50 (2%); pit bull, 1/50 (2%); and pug, 1/50 (2%). The mean (±standard deviation) age of the animals was 6.4 (±2.4) years, and the median (min–max) weight was 9.7 (4–24.5) kg. A total of 28 dogs were male, and 22 were female. The underlying cause resulting in neurological deficits in the dogs included in the study was most commonly IVDD Hansen type I, recorded in 43/50 dogs (86%), and IVDD Hansen type II, recorded in 4/50 (8%); followed by vertebral fracture in 1/50 (2%); subarachnoid diverticulum in 1/50 (2%); and spinal meningioma in 1/50 (2%), as shown in Table S1.

Breeds in the CM group were French bulldog, 10/25 (40%); mongrel, 6/25 (24%); cocker spaniel, 4/25 (16%); Chihuahua, 2/25 (8%); pinscher, 2/25 (8%); and beagle, 1/25 (4%). The mean age of the animals was 6.7 (min 3–max 11) years, and the mean weight was 10.7 (min 4.0–max 24.5) kg. Seven dogs were intact males, and six were neutered males; two dogs were intact females, and ten were spayed females. The most common underlying cause for CM was IVDD Hansen type I, recorded in 21/25 dogs (84%). IVDD Hansen type II was recorded in 3/25 (12%) dogs, and spinal neoplasia was recorded in 1/25 (4%) dogs.

Breeds in the TLM group were mongrel, 8/25 (32%); French bulldog, 5/25 (20%); Maltese, 4/25 (16%); Pekingese, 3/25 (12%); Jack Russell, 2/25 (8%); West Highland terrier, 1/25 (4%); pit bull, 1/25 (4%); and pug 1/25 (4%). The mean age of the animals was 6.1 (2–10) years, and the mean weight was 9.9 kg (5.5–23.0). Six dogs were intact males, nine males were neutered, one dog was an intact female, and nine females were spayed. The most common underlying cause for TLM was IVDD Hansen type I, recorded in 22/25 (88%) dogs, and IVDD Hansen type II was recorded in 1/25 (4%) dogs, vertebral fracture in 1/25 (4%), and subarachnoid diverticulum in 1/25 (4%).

During anaesthesia and throughout the 30 min Pdi measurement procedure, the dogs were allowed to breathe spontaneously, and none required mechanical ventilation.

### 3.2. Neurological Grading (MFS) Evaluation and Pdimax Association

Each dog underwent a neurological examination and was assigned a grade from 1 to 5 based on the Modified Franken Scoring (MFS) scale (0–5). For statistical analysis, these grades were converted into fractions (1/5 = 0.2, 2/5 = 0.4, 3/5 = 0.6, 4/5 = 0.8, 5/5 = 1). In the CM group, four dogs (4/25, 16%) were graded as 1/5 (0.2), seven dogs (7/25, 28%) as 2/5 (0.4), eight dogs (8/25, 32%) as 3/5 (0.6), and six dogs (6/26, 24%) as 4/5 (0.8).

In the TLM group, one dog (1/25, 4%) was graded as 1/5 (0.2), three dogs (3/25, 12%) as 2/5 (0.4), eight dogs (8/25, 32%) as 3/5 (0.6), six dogs (6/25, 24%) as 4/5 (0.8), and seven dogs (7/25, 28%) as 5/5 (1.0).

The mean values of Pdimax10, 20, and 30 in each group (TLM and CM) were examined for a possible correlation; a non-significant negative correlation was observed between Pdimax and MFS in the CM group (*p* = 0.1). However, a significant negative correlation between Pdimax and MFS was identified in the TLM group (*p* = 0.046).

The mean values of Pdimax for the CM and TLM groups, derived from Pdimax10, Pdimax20, and Pdimax30 within each group, were analysed for potential correlations. In the CM group, a non-significant negative correlation was observed between Pdimax and MFS (*p* = 0.1). However, a significant negative correlation was identified between Pdimax and MFS in the TLM group (*p* = 0.046), Table S2.

### 3.3. Arterial Gases PaO2 and PaCO2

Arterial gas sampling, before and after Pdimax measurement, was available for some of the dogs included in the study. An arterial blood sample was available for only 10/25 (40%) dogs in the TLM group and for 15/25 (60%) in the CM group; thus, only descriptive statistics were extruded due to missing values.

In the TLM group, PaO2 and PaCO2 was measured in 10 dogs. The mean PaO2_0_ was 467.9 mmHg, and the mean PaCO2_0_ was 51.4 mmHg. The mean PaO2_30_ was 466.3 mmHg, and the mean PaCO2_30_ was 50.9 mmHg.

In the CM group, PaO2 and PaCO2 were measured in 14 dogs. The mean PaO2_0_ was 439.5 mmHg, and the mean PaCO2_0_ was 51.9 mmHg. The mean PaO2_30_ was 400.6 mmHg, and the mean PaCO2_30_ was 54.3 mmHg, as shown in Table S2.

### 3.4. Differences in Pdimax Between CM and TLM

In the TLM group, Pdimax10 was 9.4 mmHg ± 6.1, and in the CM group, it was 7.2 mmHg ± 4.6 (*p* = 0.167). Pdimax20 was 10.2 mmHg ± 5.8 in the TLM group and 8 mmHg ± 5.1 in the CM group (*p* = 0.155). Pdimax30 was 9.2 mmHg ± 5.5 in the TLM group and 8.1 mmHg ± 5.5 in the CM group (*p* = 0.479). Pairwise comparisons revealed a significant difference only in the TLM group between Pdimax20 and Pdimax30 (*p* = 0.012), Table S2.

## 4. Discussion

Dogs suffering from CM or TLM often require multiple anaesthesia sessions for diagnostic and surgical procedures [28]. The respiratory function evaluation of these patients is critical to minimize perianaesthetic complications and reduce morbidity and mortality [28,29]. Respiratory distress, identified either through arterial blood gas measurements or clinical manifestations, is commonly observed in dogs with CM, a condition frequently seen in both human and veterinary medicine [9,11,12,16,17,20,21,22,24,25,26,30,31,32,33]. However, the veterinary literature on diaphragmatic dysfunction in dogs with CM or TLM is scarce. In one observational case-control study, diaphragmatic dysfunction was compared between a control group and CM-affected dogs, and statistically non-significant differences were reported. It was concluded that diaphragmatic paresis could not be easily detected using radiography, M-mode ultrasonography, or fluoroscopy [25]. In the study of Drury et al. (2020) [25], diaphragmatic dysfunction was also reported in the control group, consisting of both healthy dogs and dogs suffering from TLM, in addition to the group of dogs with cervical spinal disorders. This finding aligns with our observations that diaphragmatic contractility impairment may be present in dogs with TLM. Our main hypothesis was based on the expectation that Pdimax values would be lower in the CM group, due to diaphragmatic contractility impairment caused by direct phrenic nerve involvement, compared to the TLM group. However, the results did not support this assumption, and we rejected our main hypothesis because the Pdimax values in CM-affected patients were non-significantly lower than those in TLM-affected dogs.

Measuring the Pdi in dogs provides valuable insights into diaphragmatic function and respiratory processes. The methodology must strike a balance between accuracy, consistency, and minimizing confounding factors such as anaesthesia-induced changes in breathing. Maintaining a consistent position, such as lying on one side, is essential for ensuring stable Pdi values. Diaphragmatic function may be influenced by body posture, and recumbency can alter the distribution of pressures within the chest and abdomen. By keeping the same position throughout testing, these variations are minimised. Anaesthesia itself may affect respiration, including diaphragm function, EtCO2 levels, and central respiratory drive. Limiting Pdi measurements to a short duration (e.g., 30 min) reduces the likelihood of significant anaesthetic effects interfering with the results in ISO-anaesthetised dogs [4,34].

In humans, respiratory distress resulting from direct phrenic nerve involvement after CM injury, or secondary to respiratory infections, is a leading cause of death in patients with spinal cord injuries [12,20,21,22]. In the present study, none of the dogs suffered from coexisting respiratory infection. In cases of complete spinal cord injury above the third cervical segment, high mortality rates are reported, and mechanical ventilation is necessary for adequate ventilation due to trauma to respiratory centres, leading to diaphragmatic paralysis. Oxygen supplementation is also recommended for patients with lesions caudal to C3 [12,20,21,22]. Only a few dogs in our study presented with CM above the third cervical segment, preventing a statistical comparison with dogs diagnosed with CM below C3.

In human medicine, a higher percentage of respiratory distress is observed in patients with high thoracic spinal cord lesions T1–T6 (51.5%) compared to low thoracic lesions T7–T12 (34.5%) [22]. Moreover in human medicine, respiratory function in patients with thoracolumbar lesions below T12 can resemble that of healthy individuals [20]. Although there are no known direct motor pathways connecting the respiratory centres to the diaphragm through the thoracic nerves, diaphragmatic contractility impairment in TLM in humans is attributed to intercostal muscle paresis or paralysis. This disruption destabilises the rib cage and impairs the mechanical efficiency of diaphragmatic contraction, leading to respiratory dysfunction [22]. On the other hand, the veterinary literature on respiratory impairment in dogs with TLM is limited.

Despite the fact that it remains unclear if intercostal paresis or paralysis in dogs with TLM could lead to similar rib cage destabilization as observed in humans, it is plausible that disruption of intercostal muscle innervation may contribute to the lower Pdimax values observed in the dogs in our study compared to those reported in healthy dogs [4].

In our study, in dogs in the TLM group, Pdimax initially increases at the first and second time points, recorded at 10 and 20 min. However, by the third time point (Pdimax30), a statistically significant decrease compared to the earlier measurements is recorded. This decrease may be explained by the potential impact of intercostal muscle paresis or paralysis associated with TLM, which can result in greater rib cage instability. Additionally, the combined effects of anaesthesia-induced diaphragmatic fatigue, which tends to increase as the procedure progresses, likely contributed to the observed reduction in Pdimax values over time. These factors appear to be more pronounced in the TLM group compared to the CM group, highlighting the compounding challenges posed by TLM on respiratory distress. In the CM group, although there is a slight increase in Pdimax values over time, these differences are not statistically significant across the measured time points. This could be attributed to the fact that the intercostal muscles are likely less affected in dogs with CM compared to those with TLM, resulting in minimal disruption to rib cage anatomy. Therefore, the Pdimax in the CM group is presumed to be influenced primarily by the focal spinal cord defect, without the involvement of rib cage instability caused by intercostal muscle paresis or paralysis. As a result, the Pdimax remains stable in the CM group throughout the first 30 min of anaesthesia.

There is limited information in veterinary medicine regarding diaphragmatic contractility impairment after direct trauma to the cervical spine, leading to respiratory distress. In a recent retrospective study of French bulldogs, it was concluded that 19.6% of dogs presented for IVDD management experienced respiratory compromise. However, diaphragmatic paresis or paralysis was not investigated, and respiratory compromise was primarily attributed to brachycephalic obstructive airway syndrome (BOAS)-associated issues, which led to oxygen supplementation and mechanical ventilation [24]. The findings of the present study suggest that respiratory distress in dogs with cervical myelopathy (CM) or thoracolumbar myelopathy (TLM) may be linked to impaired diaphragmatic contractility. In the absence of any underlying thoracic or abdominal cavity pathology, the dogs included in the present study were classified as ASA II. The impairment of diaphragmatic contractility reduces thoracic wall expansion. This impaired expansion results in hypoventilation, and the decrease in tidal volume causes hypercapnia [2,8]. It remains unknown whether the chest wall compliance of CM- or TLM-affected and BOAS-affected dogs could result in differences in airway pressure measurements compared to healthy brachycephalic dogs. Further investigation is needed to explore this possibility. The Pdimax values recorded at all time points for both the CM and TLM groups were lower than those reported in healthy dogs anaesthetised with the same anaesthetic protocol [4]. Particularly in brachycephalic dogs, the combination of breed-related BOAS and diaphragmatic contractility impairment could possibly result in severe respiratory distress. However, in our study, none of the brachycephalic dogs required emergency intubation or BOAS surgery.

Isoflurane was selected as the agent to maintain general anaesthesia, as it is known to be a mild depressant of diaphragmatic function. According to the literature, diaphragmatic function was found to be statistically non-significantly affected in ISO-anaesthetised dogs following mechanical stimulation of the phrenic nerve [34]. Additionally, in in vitro studies in rats, it was concluded that ISO has minimal impact on diaphragmatic motility [35]. In a study by Pavlidou et al. (2013) [4], the impact of different anaesthetic protocols on Pdimax was investigated, and ISO was found to have a statistically significant weak suppressive effect on Pdimax in dogs.

To minimize the risk of respiratory compromise due to underlying pathology, only ASA II dogs were included in this study [8]. Overweight patients were also excluded, as obesity has been shown to affect respiratory function in both humans and dogs [36,37,38,39]. In humans, an increased body mass index is associated with reduced breathing volume [36,37,38]. Similarly, it has been reported that the inspiratory volume per kilogram of body mass is lower in obese dogs [39].

Respiratory function in dogs can also be assessed through arterial blood gas analysis [2,8]. In a prospective cohort study in dogs undergoing cervical decompressive surgery for IVDD, it was reported that perioperative subclinical hypoventilation (PaCO2 ≥ 45 mmHg) was noted [23]. In our study, arterial blood gas samples were collected from anaesthetised dogs under ISO in 100% oxygen, both before and after Pdimax measurements, to evaluate PaCO2 and PaO2 levels. In the TLM group, arterial blood samples were obtained from 10 out of 25 dogs (40%). Before the starting of the Pdimax, 7 out of 10 dogs (70%) had a PaCO2 ≥ 45 mmHg, increasing to 9 out of 10 dogs (90%) at 30 min. In the CM group, arterial blood samples were collected from 14 out of 25 dogs (56%). Similarly, in this group, 9 out of 14 dogs (64%) had a PaCO2 ≥ 45 mmHg before the start of Pdimax measurements, rising to 10 out of 14 dogs (71%) at 30 min. These findings indicate the presence of hypoventilation in both the TLM and CM groups, observed consistently before and 30 min into Pdimax measurements in anaesthetised dogs.

Higher MFS scoring in surgically treated dogs with CM has also been associated with higher estimated PaCO2 levels [23]. A negative correlation between Pdimax and the severity of symptoms, as assessed by the MFS, was observed in the TLM group but not in the CM group. Dogs in the TLM group with higher MFS scoring tended to have lower Pdimax values. This could be attributed to the fact that the dogs in the TLM group had a mean severity score of 3/5 (0.6) compared to those in the CM group, which had a mean severity score of 2/5 (0.4). There is limited information on the correlation between diaphragmatic contractility impairment, neurological severity (MFS), and PaCO2 values. Exploring any potential relationship between MFS and diaphragmatic contractility in dogs with CM or TLM would be of significant interest, as MFS can be assessed and evaluated by clinicians in everyday clinical practice. Further studies are needed to validate and strengthen these findings.

The first limitation of this study was the absence of a control group of healthy dogs, which would have allowed for a direct comparison of Pdimax values between the control group and CM group and also with the TLM group. Another limitation is the lack of arterial blood samples from all patients, which resulted in the use of descriptive statistics for evaluation. Additionally, potential differences between lesions dorsal and caudal to C3, as well as between upper and lower thoracic lesions, or comparison between Pdimax values across these subgroups, could not be investigated due to the limited sample size. Taking into consideration these observations, future researchers could further investigate this topic.

## 5. Conclusions

According to the findings of our study there, are statistically non-significant differences in diaphragmatic contractility values between dogs with CM and TLM under ISO anaesthesia. Additionally, the negative correlation observed in the TLM group between the MFS and Pdimax indicates an association between neurological severity and diaphragmatic contractility impairment that needs further investigation. The presence of hypoventilation in both CM- and TLM-affected dogs supports the notion of respiratory dysfunction in dogs affected by focal myelopathy in both the cervical and thoracolumbar spinal cord. The importance of respiratory support is highlighted by the findings of this study, demonstrating its necessity not only for dogs with CM but also for those affected by TLM.

Further prospective studies are needed to clarify and expand our understanding of the role of the diaphragm in respiratory distress in dogs with CM and TLM.

## Data Availability

The datasets generated and analysed during the study are available from the corresponding author upon request.

## Supplementary Materials

The following supporting information can be downloaded at: https://www.mdpi.com/article/10.3390/ani15020147/s1, Table S1: Demographics; Table S2: This table summarises the results of Transdiaphragmatic pressure (Pdimax) in the different recorded timepoints, the Modified Frankel Scoring results (MFS) and arterial blood gas parameters (PaCO2 and PaO2) at the beginning and at the ending of the measurements in groups TLM and CM and the values are presented as group average.
