# Supplementary material for: Differences in Transdiaphragmatic Pressure of Dogs Suffering from Cervical or Thoracolumbar Myelopathy Anaesthetised with Isoflurane
Source: Animals (Basel). 2025 Jan 9;15(2):147. doi: 10.3390/ani15020147 (PMC11758306; doi:10.3390/ani15020147)
Supplement: Supplementary file 1 [file animals-15-00147-s001.zip › animals-3358770-supplementary.pdf]

Patient Number	Age years	Sex (Male/ Female-Neutered/Spayed)	Weight (Kg)	Breed	Diagnosis
TLM 1	8,5	F	6,8	Maltese	IVVD Type I L2-L3
TLM 2	5,0	F spayed	6,7	Mongrel	IVVD Type I T13-L1
TLM 3	6,0	F spayed	17,0	Mongrel	IVVD Type I L2-L3
TLM 4	6,0	M	8,0	Pekignese	IVVD Type II L2-L3
TLM 5	7,0	M	6,5	Westie	IVVD Type I L1-L2
TLM 6	5,5	F spayed	18,0	Mongrel	IVVD Type I T11-T12
TLM 7	3,5	F spayed	9,2	French Bulldog	IVVD Type I T12-T13
TLM 8	3,5	F spayed	7,4	Mongrel	IVVD Type I L2-L3
TLM 9	4,0	F spayed	14,6	French Bulldog	IVVD Type I L1-L2
TLM 10	2,0	M	10,8	French Bulldog	IVVD Type I L2-L3
TLM 11	8,0	F Spayed	23,0	Pit Bull	IVVD Type I T12-T13
TLM 12	7,0	M neutered	6,0	Pekignese	IVVD Type I T12-T13
TLM 13	6,0	M neutered	7,0	Jack Russel	IVVD Type I T12-T13
TLM 14	3,5	M neutered	10,0	French Bulldog	IVVD Type I T12-T13
TLM 15	7,0	M neutered	8,0	Mongrel	IVVD Type I L2-L3
TLM 16	5,0	M	8,0	Mongrel	Vertebral Fracture L2-L3
TLM 17	7,0	M neutered	11,6	Mongrel	IVDD Type I L1-L2
TLM 18	5,0	F spayed	6,6	Maltese	IVVD Type I L2-L3
TLM 19	4,0	M neutered	13,2	Mongrel Beagle	IVVD Type I T11-T12
TLM 20	3,0	M neutered	7,0	Jack Russel	IVVD Type I T12-T13
TLM 21	9,0	M	7,5	Maltese	IVVD Type I T13-L1
TLM 22	10,0	M	10,0	Pug	IVVD Type I T11-T12
TLM 23	9,0	F spayed	9,0	Mongrel Pincher	IVVD Type I L2-L3
TLM 24	8,0	M neutered	5,5	Maltese	IVVD Type I L1-L2
TLM 25	10,0	M neutered	11,0	French Bulldog	Subarachnoid Diverticulum T8
CM 1	3,0	M	11,0	French Bulldog	IVVD Type I C3-C4
CM 2	3,0	M spayed	13,0	French Bulldog	IVVD Type I C4-C5
CM 3	6,5	M	9,4	Mongrel	IVVD Type I C3-C4
CM 4	5,0	F neutered	13,5	Cocker Spaniel	IVVD Type I C3-C4
CM 5	3,5	F neutered	9,0	French Bulldog	IVVD Type I C3-C4
CM 6	10,0	M	13,5	Cocker Spaniel	IVVD Type II C3-C4
CM 7	5,0	M	12,0	French	IVVD Type I C3-C4

				Bulldog	
CM 8	8,0	F neutered	10,8	French Bulldog	IVVD Type I C2-C3
CM 9	7,0	F neutered	12,0	Cocker Spaniel	IVVD Type I C2-C3
CM 10	6,0	F	5,4	Chihuahua	IVVD Type I C3-C4
CM 11	7,0	M	4,0	Chihuahua	IVVD Type I C2-C3
CM 12	10,0	M	8,4	Pincher	IVVD Type II C3-C4
CM 13	5,5	F neutered	11,0	French Bulldog	IVVD Type I C3-C4
CM 14	8,0	F neutered	7,0	Mongrel	IVVD Type I C2-C3
CM 15	9,0	M spayed	24,5	Cocker Spaniel	IVVD Type I C6-C7
CM 16	7,0	M spayed	14,3	Mongrel	IVVD Type I C3-C4
CM 17	7,0	F	5,0	Pincher	IVVD Type I C4-C5
CM 18	4,0	F neutered	6,7	Mongrel	IVVD Type I C2-C3
CM 19	11,0	M	9,0	Mongrel	IVVD Type I C5-C6
CM 20	9,0	F neutered	13,0	French Bulldog	Meningioma A2-A3
CM 21	11,0	M spayed	7,3	Mongrel	IVVD Type I C3-C4
CM 22	6,0	M spayed	11,8	French Bulldog	IVVD Type I C3-C4
CM 23	8,0	M spayed	15,0	Beagle	IVVD Type II C2-C3
CM 24	4,0	F neutered	10,5	French Bulldog	IVVD Type I C2-C3
CM 25	3,0	F neutered	10,3	French Bulldog	IVVD Type I C2-C3

Table S1. Demographics

	**Pdi max 10 mmHg**	**Pdi max 20 mmHg**	**Pdi max 30 mmHg**	**MFS**		**PaCO2 0 mmHg**	**PaO2 0 mmHg**	**PaCO2 30 mmHg**	**PaO2 30 mmHg**
**TLM GROUP AVERAGE**	**9,39**	**10,24**	**9,25**	**3/5**	**0,6**	51,37	467,92	50,89	466,29
**CM GROUP AVERAGE**	**7,25**	**7,99**	**8,13**	**2/5**	**0,4**	51,91	439,55	54,35	400,63

Table S2.

This table summarises the results of Transdiaphragmatic pressure (Pdimax) in the different recorded timepoints, the Modified Frankel Scoring results (MFS) and arterial blood gas parameters (PaCO2 and PaO2) at the beginning and at the ending of the measurements in groups TLM and CM and the values are presented as group average.
